# Analysis of Collapse–Snapback Phenomena in Capacitive Micromachined Ultrasound Transducers

**DOI:** 10.3390/mi16020160

**Published:** 2025-01-29

**Authors:** Chloé Halbach, Veronique Rochus, Jan Genoe, Xavier Rottenberg, David Cheyns, Paul Heremans

**Affiliations:** 1 Interuniversity Microelectronics Centre, Kapeldreef 75, 3001 Leuven, Belgium; veronique.rochus@imec.be (V.R.); jan.genoe@imec.be (J.G.); xavier.rottenberg@imec.be (X.R.); david.cheyns@imec.be (D.C.); paul.heremans@imec.be (P.H.); 2 Department of Electrical Engineering, KU Leuven, Kasteelpark Arenberg 10, 3001 Leuven, Belgium

**Keywords:** capacitance–voltage characteristics, dynamic capacitance, electrostatic pull-in, LCR meter, Laser Doppler Vibrometer, COMSOL Multiphysics simulations

## Abstract

The pull-in and pull-out voltages are important characteristics of Capacitive Micromachined Ultrasound Transducers (CMUTs), marking the transition between conventional and collapse operation regimes. These voltages are commonly determined using capacitance–voltage (C-V) sweeps. By modeling the operating conditions of an LCR meter in COMSOL Multiphysics^®^, we demonstrate that the measured capacitance comprises both static and dynamic capacitances, with the dynamic capacitance causing the appearance of a peak in the effective C-V curve. Furthermore, Laser Doppler Vibrometer (LDV) measurements and electromechanical simulations indicate the occurrence of collapse–snapback phenomena during the C-V sweeps. This study, through advanced simulations and experimental analyses, demonstrates that the transient membrane behavior significantly affects the apparent capacitance–voltage characteristics of electrostatically actuated Micro-Electromechanical Systems (MEMS).

## 1. Introduction

The pull-in (collapse) and pull-out (snapback) voltages are critical parameters that delineate the operational modes of electrostatically actuated Micro-Electromechanical Systems (MEMS), such as RF MEMS switches [1] and Capacitive Micromachined Ultrasound Transducers (CMUTs). Lower pull-in voltages are particularly desirable as they reduce the energy required for efficient operation in both RF MEMS switches and CMUTs. In CMUTs specifically, these voltages define the boundary between the conventional and collapse operation regimes [2,3,4,5]. Previous research indicates that the collapse mode provides higher acoustic output pressure, ranging from 25 to 50 kPa/V, compared to the conventional mode, which typically ranges from 10 to 20 kPa/V [4,6,7]. However, the collapse mode is more susceptible to dielectric charging, which can adversely affect their long-term reliability [8]. Accurately determining the voltage limits between the conventional and collapse operation regimes is therefore crucial for optimizing performance and ensuring device operation in the desired mode.

The pull-in phenomenon, resulting in the abrupt collapse of a suspended structure onto a fixed bottom electrode, can be categorized into static and dynamic pull-in depending on the actuation scheme. The static pull-in voltage is obtained by slowly increasing the DC voltage such that the system can be assumed to be in static equilibrium at each point in time until collapse. This quasi-static or steady-state assumption becomes invalid when transients caused by DC or AC loading cannot be ignored. For example, classical mechanics demonstrates that the dynamic pull-in voltage of an undamped clamped–clamped beam suddenly subjected to a voltage step is approximately 8% lower than the static pull-in voltage [9]. In some resonant MEMS applications, where the device is deflected by a DC voltage and actuated by an AC voltage around its natural resonance frequency to enhance transducer sensitivity, pull-in occurs at even lower voltages [10]. Specifying the conditions under which the pull-in voltage is determined is essential because the measured value is sensitive to the measurement approach and test signal.

A common method to measure the pull-in and pull-out voltages is to perform a capacitance–voltage sweep [5,11,12,13]. The pull-in voltage coincides with a sudden increase in capacitance when the voltage is gradually increased, whereas the pull-out voltage corresponds to a sharp decrease in capacitance when the voltage is gradually reduced after the membrane has been pulled in. Such measurement is generally performed with an impedance analyzer or LCR meter by applying a small AC voltage (30 mV–1 V) on top of the DC voltage at a test frequency far away from the natural resonance frequency of the device under test (DUT) [5]. Since the DC voltage is ramped up in small voltage steps compared to the pull-in voltage, the measured pull-in voltage is close to the static pull-in voltage.

Hysteresis is a common phenomenon in non-linear actuators that exhibit switching of states [14]. When the voltage is reduced after collapse, the plate will only snap back when the upward mechanical spring force exceeds both the stiction resisting motion and the downward electrostatic force. The voltage at which the membrane snaps back is called the pull-out voltage. Due to the complexity of modeling stiction, analytical solutions for determining the pull-out voltage are not readily available. Instead, semi-analytical and Finite Element Method (FEM) models can be employed to estimate this value, which generally occurs at a voltage lower than the pull-in voltage [15,16,17,18].

This work demonstrates minimal hysteresis between pull-in and pull-out for CMUTs exhibiting significant initial downward deflection and reveals that the capacitance–voltage (C-V) measurements performed near the pull-in voltage can exhibit a sharp rise followed by a sharp drop in the effective capacitance, as observed though not explained by Merbeler et al. [5] and Dew et al. [13]. At first, we show that the peak in capacitance measured with an LCR meter coincides with the collapse–snapback mode visualized with a Laser Doppler Vibrometer (LDV). Secondly, we demonstrate that the C-V sweep depends on the amplitude of the AC voltage but not on the frequency of the test signal when the latter is kept away from the natural resonance frequency of the DUT. Thirdly, we model the operating conditions of an LCR meter in COMSOL Multiphysics^®^ and show that the peak in the effective capacitance also appears in the simulations when the AC test signal is superimposed on the DC pre-stress voltage. Finally, we provide evidence that the measured capacitance can be decomposed into static and dynamic capacitances, and that the peak in capacitance is due to the dynamic part. Our objective is to provide deeper insights into the impact of collapse–snapback phenomena on the C-V characteristics of CMUTs through advanced simulations and experimental investigations.

## 2. Materials and Methods

### 2.1. Fabrication Methods

**Figure 1:**
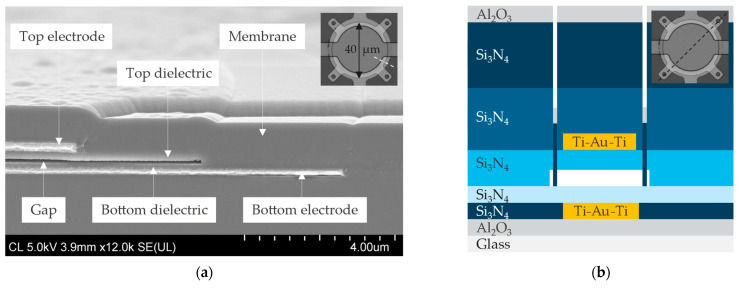
(**a**) SEM image and (**b**) schematic cross section of the fabricated CMUTs. Topographical changes around the bottom electrode are significantly smaller compared to the steps caused by the sacrificial layer and the top electrode thanks to the self-aligned lift-off process.

Figure 1 displays a SEM cross section (a), a schematic cross section (b), and a microscope top view of the CMUTs fabricated by sacrificial release on 6-inch borosilicate glass wafers (EAGLE XG^®^, Corning^®^, Corning, NY, USA). One particularity of the process flow is that the bottom electrode (200 nm Ti-Au-Ti) is embedded in dielectric grooves (200 nm Si_3_N_4_) to reduce step coverage issues in the subsequent process steps [19]. The bottom electrode is patterned with a so-called self-aligned lift-off method, which consists of dry etching trenches in a dielectric with the aid of a photoresist that is kept as the lift-off layer for the subsequent metal deposition, such that, after lift-off, the metal becomes self-aligned in the dielectric grooves with a single lithography step. The bottom dielectric (100 nm Si_3_N_4_), the gap (100 nm), and the top dielectric (200 nm Si_3_N_4_) should be as thin as possible to improve the CMUT sensitivity, while avoiding dielectric breakdown during operation [20]. The effective gap, defined in Equation (10) as the effective distance between the top and bottom electrodes, can be limited by selecting dielectrics with a high dielectric constant. Silicon nitride (ε=7) is therefore preferred above silicon dioxide (ε=4) [21].

The sacrificial layer (100 nm Cr) and the top electrode (250 nm Ti-Au-Ti) are patterned with a standard lift-off process. Then, the top electrode is covered with 500 nm PECVD Si_3_N_4_, and 3 μm diameter release holes are dry etched into the Si_3_N_4_ to access the Cr sacrificial layer. Ultimately, the wafer is immersed in chromium etchant (Chromium Etchant 1020, Transene, Danvers, MA, USA) for 3.5 h to etch away the Cr sacrificial layer. Immediately after the wet release of the membranes, we rely on a CO_2_-based critical point dryer (Automegasamdri-916B, Tousimis, Rockville, MD, USA) to prevent membrane capillary stiction. The release holes are subsequently filled with 500 nm PECVD Si_3_N_4_ and 50 nm ALD Al_2_O_3_ to seal the cavities. The additional Al_2_O_3_ prevents liquid ingress into the sealed cavities by obstructing the Si_3_N_4_ stress lines that form at the edges of the release holes during via filling. Finally, electrical contact pads are patterned by dry etching the Al_2_O_3_ and Si_3_N_4_ layers with Cl_2_/BCl_3_ and SF_6_/O_2_, respectively.

In summary, the CMUTs are made of a 200 nm Ti-Au-Ti bottom electrode, a 100 nm Si_3_N_4_ bottom dielectric, a 100 nm cavity, and a multilayered membrane consisting of 200 nm Si_3_N_4_, 250 nm Ti-Au-Ti, 1 μm Si_3_N_4_, and 50 nm Al_2_O_3_. The membrane diameter of the CMUT presented in this work is 40 µm.

### 2.2. Electrical Characterization Methods

Two methods were tested to measure the capacitance of the CMUT as a function of the applied DC bias. The first method relies on an LCR meter (Agilent E4980 with 16048A test leads, Keysight, Santa Rosa, CA, USA) to determine capacitance via impedance measurements. The C-V curve is obtained by superimposing the AC test signal, ranging from 0.5 V to 4 V, onto a DC bias swept from 0 V to 35 V in 1 V increments, ensuring the combined AC + DC voltage remains within the 42 V peak limit of the Agilent E4980 LCR meter [22]. The capacitance of the DUT is extracted using either a series Cs-Rs or a parallel Cp-Rp equivalent circuit [23]. These measurement modes are related through the dissipation factor D [-] by the equations:

(1) $$D=2\pi fC_{s}R_{s}=\;\frac{1}{2\pi fC_{p}R_{p}}$$

and

(2) $$C_{s}=C_{p}\;\left(1+D^{2}\right)$$

where $C_{s}$ [F] and $R_{s}$ [Ω] are the equivalent series capacitance and resistance, $C_{p}$ [F] and $R_{p}$ [Ω] are the equivalent parallel capacitance and resistance, and f [Hz] is the test frequency. Given that the dissipation factor of the CMUT in this study is less than 0.005 (See Section 3.2), both equivalent circuits can be used interchangeably. Moreover, the measured capacitance varies by less than 0.5% across test frequencies of 50 kHz, 100 kHz, and 1 MHz (Supplementary Figure S1). This minimal frequency dependence highlights the negligible impact of parasitic effects, enabling the DUT to be effectively modeled as an ideal capacitor.

The second method relies on a Quasi-Static Capacitance Voltage (QSCV) measurement performed with a parameter analyzer (Agilent 4156C, Keysight). The parameter analyzer applies a voltage step ΔV [V] to the DUT and measures the transient charge current I(t) [A] that flows in response to the sudden change in applied voltage [24]. Integrating this current over a measurement time ΔT [s] that covers all the transient charge current yields the amount of charge ΔQ [C] induced by the voltage step ΔV from which the capacitance C [F] can be deduced:

(3) $$C=\frac{\Delta Q}{\Delta V}=\frac{\int_{0}^{\Delta T}i\left(t\right)dt}{\Delta V}$$

As for the LCR meter setup, the C-V curves are generated by measuring the capacitance for a voltage step ΔV superimposed on a DC bias. The QSCV measurement parameters performed on capacitances of the order of 1–10 pF are reported in Table 1.

### 2.3. Electromechanical Characterization Methods

Purely optical methods, like white light interferometry, face limitations in capturing the complete membrane profile due to the optically transparent silicon nitride membrane, steep slopes, and step changes near the edges of the cavity. Therefore, we propose using a dynamic electromechanical characterization method instead. The experimental setup consists of a Laser Doppler Vibrometer (MSA-500, Polytec, Waldbronn, Germany) combined with an arbitrary waveform generator (33500B, Keysight), a high-voltage amplifier (WMA-300, Falco, Hilversum, The Netherlands), and an oscilloscope (TBS 2000B, Tektronix, Beaverton, OR, USA). A timed acquisition on the LDV, which is synchronized with the trigger of the waveform generator, monitors the relative displacement of the membrane center while the voltage applied on the DUT is increased beyond pull-in. The initial membrane deflection is then obtained by subtracting the maximum relative displacement of the membrane center from the gap expected for a perfectly flat membrane. The latter corresponds to the 100 nm sacrificial layer thickness and is measured with a stylus profilometer (DektakXT, Bruker, Billerica, MA, USA) during fabrication.

A Laser Doppler Vibrometer (MSA-500, Polytec), an arbitrary waveform generator (33500B, Keysight), a DC power supply (E36232A, Keysight), a custom-made bias tee, and an oscilloscope (TBS 2000B, Tektronix) are employed to measure the natural resonance frequency of the CMUT as a function of the applied DC bias. The bias tee superimposes the AC on the DC voltage on the CMUT side, while protecting the DC power supply and AC generator from potential damage. The same setup is used to replicate the operating conditions of the LCR meter when exciting the CMUT well below its natural resonance frequency. Scans with a grid point spacing of 3.5 µm are acquired to visualize the membrane motion under specific biasing conditions.

### 2.4. Analytical Methods

The resonance frequency $f_{r}$ [Hz] of a CMUT operating in conventional mode is generally expressed as:

(4) $$f_{r}=\;\frac{1}{2\pi }\;\sqrt{\frac{k_{eff}}{m_{eff}}}$$

where $k_{eff}$ [N/m] is the effective spring constant, and $m_{eff}$ [kg] is the effective mass of the membrane. The effective spring constant is influenced by both the residual stress in the membrane [25] and the externally applied DC voltage [26]. To better understand the natural behavior of the system, the natural resonance frequency $f_{0}$ [Hz] of a circular clamped plate without residual stress and DC voltage can be expressed as:

(5) $$f_{0}=\;\frac{10.2158}{2\pi R^{2}}\;\sqrt{\frac{D}{\rho t}}$$

where R [m], t [m], and ρ [kg/m^3^] are the radius, thickness, and density of the plate, respectively [27]. For an isotropic monolayered circular plate, the flexural rigidity D [N·m] is determined by:

(6) $$D=\;\frac{Et^{3}}{12\;\left(1\;-\;\nu^{2}\right)}$$

where *E* [Pa] and ν [-] are the Young’s modulus and Poisson’s ratio of the plate, respectively. For multilayered plates, the flexural rigidity must account for the neutral bending plane [28]. The application of a DC bias voltage $V_{DC}$ [V] induces a spring softening effect. Assuming a piston-like motion of the plate, this softening effect modifies the resonance frequency $f_{DC}$ [Hz] as follows:

(7) $$f_{DC}=\;f_{0}\;\sqrt{\;\frac{1-3x_{n}}{1-x_{n}}}$$

where $x_{n}$ [-] represents the normalized displacement of the plate relative to the effective gap height [26]. This equation assumes that $x_{n}$ approaches 1/3 as $V_{DC}$ approaches the pull-in voltage $V_{PI}$ [V], so that:

(8) $$\frac{V_{DC}}{V_{PI}}=\;\sqrt{\;\frac{27}{4}x_{n}{\left(1-x_{n}\right)}^{2}}$$

At the pull-in voltage, the system loses the ability to maintain a stable resonant mode, causing both the effective spring constant and resonance frequency to approach zero. However, when the CMUT transitions from conventional to collapse mode, its resonant behavior changes substantially due to the additional constraint from the collapsed region. As a result, the resonance frequency jumps to a significantly higher value in the collapse mode compared to the conventional mode [4].

The pull-in voltage $V_{PI}$ [V] is the critical voltage at which the downward electrostatic force overcomes the upward mechanical restoring force opposing the movement of a parallel-plate electrostatic actuator. It is analytically determined by:

(9) $$V_{PI}=\sqrt{\frac{8\;k\;t_{eff}^{3}}{27\;\varepsilon_{0}\;A}}$$

where $\varepsilon_{0}$ [F/m] is the vacuum permittivity, $t_{eff}$ [m] is the effective gap between the movable top electrode and the fixed bottom electrode, A [m^2^] is the overlapping area between the electrodes, and k [N/m] is the spring constant of the top plate [14]. The effective gap is defined as:

(10) $$t_{eff}\;=t_{gap}+\;\frac{\left(t_{top,ins}\;+\;t_{bot,ins}\right)}{\varepsilon_{ins}}$$

where $t_{gap}$ [m] is the cavity height; $t_{top,ins}$ [m] and $t_{bot,ins}$ [m] are the top and bottom insulator thicknesses, respectively; and $\varepsilon_{ins}$ [-] is the dielectric constant. The spring constant for a circular plate with a fixed circumference can be expressed as [29]:

(11) $$k=\;\frac{192\;\pi \;D}{R^{2}}$$

It is important to note that Equation (9) assumes a piston-like motion of the top plate, neglecting the parabolic displacement of a suspended structure with fixed edges. Consequently, this equation tends to overestimate the pull-in voltage compared to numerical methods that account for non-uniform gaps. For a circular CMUT, a more accurate approximation of the pull-in voltage is obtained by multiplying Equation (9) by a factor of 0.7 [30]. This correction factor allows for an accurate prediction of the pull-in voltage, provided that the membrane does not suffer from significant initial deflection.

### 2.5. Simulation Methods

**Figure 2:**
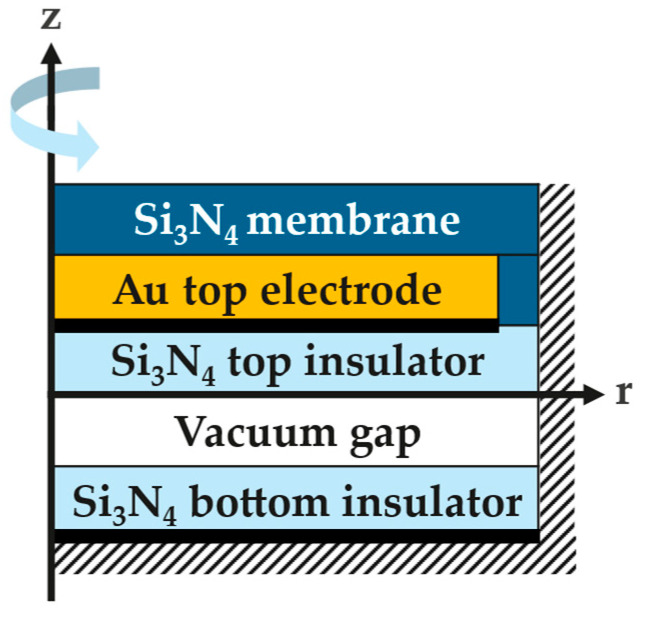
Schematic of the 2D axisymmetric CMUT model implemented in COMSOL.

The design parameters modeling the CMUT in COMSOL Multiphysics^®^ version 6.1 are schematized in Figure 2 and detailed in Table 2 and Table 3. The model consists of a 2D axisymmetric clamped Si_3_N_4_ membrane embedding a gold top electrode, a moving mesh for the vacuum gap, and a fixed Si_3_N_4_ bottom dielectric with an infinitesimally thin bottom electrode. The top electrode, 3 µm smaller in radius than the membrane, is grounded, while the bottom electrode is subjected to an applied voltage. The electrostatic force acting on the membrane is automatically implemented through the Multiphysics interface, which couples the Solid Mechanics node with the Electrostatics node. This force pulls the membrane towards the substrate when a voltage difference is applied between the electrodes. A contact pair with an automatic penalty factor and contact pair offset of 1 nm is introduced to allow the membrane to collapse onto the bottom dielectric while avoiding convergence problems with the deforming mesh [31].

Upward or downward initial membrane deflection can arise from various factors, including the inherent curvature of underlying layers, a pressure gradient acting across the membrane, and residual stress resulting from the fabrication process [32]. In this study, the membrane is deposited on a flat substrate (Figure 1a) and experiences a pressure gradient of approximately 1 atm when exposed to ambient air, as the vacuum cavity is sealed with Si_3_N_4_ and Al_2_O_3_ at 0.01 atm. COMSOL simulations indicate that this pressure gradient, exerting a downward force on the membrane’s top surface, causes a center deflection of only −5 nm (See Section 3.1). Consequently, the substantial downward deflection of −80 nm, observed experimentally in this work, is primarily attributed to residual stress.

The experimentally measured deflection profile can be implemented in COMSOL as a prescribed displacement to back-calculate the residual stress and evaluate its impact on the pull-in voltage and resonance frequency. The center deflection $w_{0}$ [m] is determined by subtracting the maximum displacement range of the membrane center from the sacrificial layer thickness, which is 100 nm in this study (see Section 2.3). The prescribed displacement in the vertical direction w [m] is then expressed as a function of the radial position r [m], as follows:

(12) $$w\left(r\right)=w_{0}\left(1-{\left(\frac{r}{R}\right)}^{2}\right)$$

This is based on the assumption that the deflection is primarily influenced by biaxial residual stress [33].

**Figure 3:**
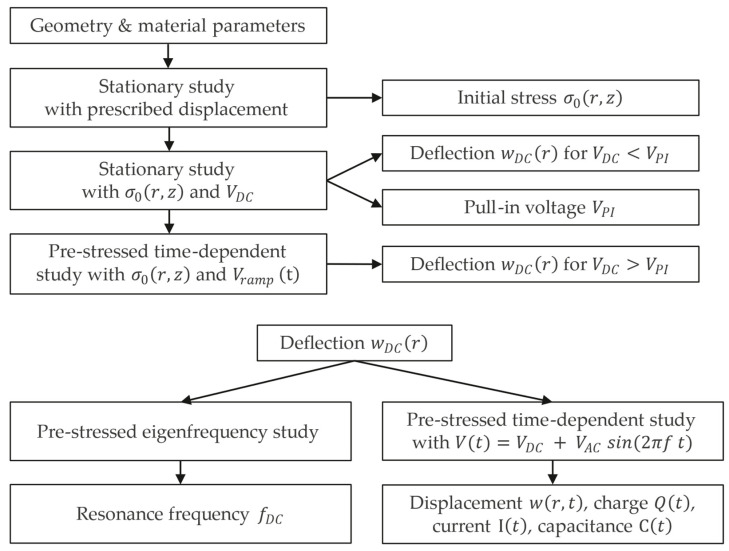
Flowchart of the studies implemented in COMSOL.

Figure 3 summarizes the studies implemented in COMSOL to simulate the pull-in voltage $V_{PI}$ [V], the resonance frequency $f_{DC}$ [Hz], and the capacitance C [F] of the CMUT experiencing substantial initial downward deflection. A custom LiveLink for MATLAB^®^ R2020a script automates the sequential execution of these studies and facilitates the export of relevant data. The stress distribution $\sigma_{0}\left(r,z\right)$ [N/m^2^], obtained from the stationary study with the prescribed displacement defined by Equation (12), is used as initial stress for subsequent studies. The pull-in voltage is identified as the maximum voltage before the membrane collapses onto the bottom dielectric. The membrane deflection $w_{DC}\left(r\right)$ for DC voltages below pull-in is readily obtained with a stationary study. However, near pull-in, a time-dependent study with a gradual voltage increase is necessary to avoid convergence issues that arise from a steeper increase in membrane displacement. This ramp voltage $V_{ramp}\left(t\right)$ [V] is defined as:

(13) $$V_{ramp}\left(t\right)=\;V_{0}+\left(V_{DC}-V_{0}\right)\;sin\left(2\pi f\;t\right)$$

where $V_{0}$ [V] is a start voltage below the pull-in voltage, $V_{DC}$ [V] is the target DC voltage above pull-in, and f=5 kHz is the ramp frequency. The membrane deflection at a specific DC voltage is then used as the initial condition for an eigenfrequency study or for a time-dependent study in which an AC voltage is superimposed on the DC voltage, such that the total time-dependent voltage $V\left(t\right)$ [V] is determined by:

(14) $$V\left(t\right)=V_{DC}\;+\;V_{AC}\;sin\left(2\pi f\;t\right)$$

where f [Hz] is the frequency of the AC voltage with amplitude $V_{AC}$ [V].

We propose two approaches to compute the capacitance from simulations. The first method mimics the operation principle of an LCR meter by simulating an impedance measurement. When a sinusoidal test voltage is applied to a DUT, its complex impedance Z [Ohm] is determined by:

(15) $$Z=\frac{V_{rms}}{I_{rms}}e^{j\theta }$$

where $V_{rms}$ [V] and $I_{rms}$ [A] are the RMS voltage and current, respectively, and θ [rad] is the phase difference between the voltage and the current. The capacitance C [F] of an ideal capacitor is expressed as:

(16) $$C=\frac{1}{\omega X_{c}}=\frac{1}{2\pi fX_{c}}=\frac{1}{2\pi f\;Im\left(Z\right)}$$

where $X_{c}=Im\left(Z\right)=1/\left(\omega C\right)=1/\left(2\pi fC\right)$ [Ω] is the reactance of the capacitor, and f [Hz] is the frequency of the AC test voltage. Substituting Equation (15) into (16) yields the following expression for the capacitance:

(17) $$C=\frac{I_{rms}}{2\pi f\;V_{rms}\;sin\left(\theta \right)}$$

The RMS voltage is computed after subtracting the DC component from the applied voltage in Equation (14). The current, $I\left(t\right)$ [A], from which $I_{rms}$ is derived, is obtained by taking the time derivative of the charge $Q\left(t\right)$ [C], as this quantity is not directly provided by the electrostatics model in COMSOL, while θ [rad] is calculated as the phase difference between the zero crossings of the current and voltage after subtracting the DC voltage offset.

The second method evaluates the capacitance based on a small-signal model and geometrical changes of the membrane. The measured capacitance C [F] can be decomposed into static and dynamic components, as follows:

(18) $$C=\frac{dQ}{dV}=\frac{dQ}{dt}\frac{dt}{dV}=\frac{d\left(C_{stat}\;V\right)}{dt}\frac{dt}{dV}=C_{stat}+\;V\;\frac{dC_{stat}}{dt}\frac{dt}{dV}=\;C_{stat}\;+\;C_{dyn}$$

The static capacitance $C_{stat}$ [F] is calculated from the static membrane profile for a specific DC bias as:

(19) $$C_{stat}=\iint_{A}\frac{\varepsilon_{0}}{t_{eff}\;+\;w}dA=\overset{R_{elec}}{\underset{0}{\int }}\frac{2\pi \varepsilon_{0}}{t_{eff}\;+\;w\left(r\right)}r\;dr$$

where $t_{eff}$ [m] is the effective gap between the bottom and top electrodes; w [m] the vertical displacement component, which is a function of the radial position r [m] along the membrane; and $R_{elec}$ [m] the radius of the overlapping electrodes. The dynamic capacitance $C_{dyn}$ [F] accounts for the transient membrane displacement generated by the AC load and is determined by:

(20) $$C_{dyn}\equiv \;V\;\frac{dC_{stat}}{dt}\frac{dt}{dV}$$

The measured capacitance for various DC and AC voltages is then computed as the sum of the static capacitance and the root mean square of the dynamic capacitance:

(21) $$C=\;C_{stat}\;+\;RMS\left\{C_{dyn}\right\}$$

## 3. Results and Discussion

### 3.1. Validation of the COMSOL Model

Table 4 summarizes the resonance frequency and pull-in voltage for the analytical model based on Equations (5)–(11), various COMSOL simulations, and experimental characterizations using an LDV and an LCR meter. A relative error of 1% between the analytical and numerical simulations validates the COMSOL model for a CMUT with full top-electrode coverage and no initial deflection.

To better replicate the actual design of the fabricated CMUT (Figure 1a), the COMSOL model is refined with a top electrode that is 3 µm shorter than the membrane radius. The resonance frequency with full top-electrode coverage is approximately 2% lower than with 85% coverage, due to the higher density of Au compared to Si_3_N_4_. Similarly, the pull-in voltage with full top-electrode coverage is slightly lower than with partial coverage, as it is inversely proportional to the square root of the overlapping area between the electrodes. However, the difference is limited to 2%, since the electrostatic force is about three times smaller near the membrane edge compared to the center.

**Figure 4:**
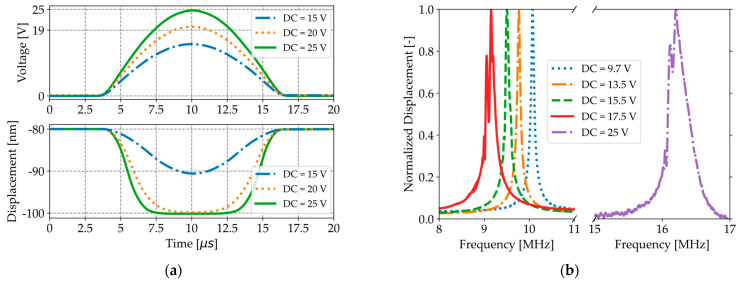
(**a**) LDV measurements of the relative displacement of the membrane center, corrected for an initial center deflection of −80 nm, as the voltage is ramped up and down between 0 V and a maximum DC value. (**b**) Resonance frequency measurements performed in air with an LDV for various DC voltages and a fixed AC amplitude of 50 mV.

LDV measurements in Figure 4a, which are corrected for an initial deflection of −80 nm and track the relative displacement of the membrane center, show that the maximum displacement of the membrane center saturates at −100 nm when the DC voltage exceeds the pull-in and pull-out voltages (19 V), as the membrane center remains in contact with the substrate in this voltage range.

The natural resonance frequency of a CMUT is the frequency at which the membrane experiences the greatest deformation due to an external excitation. The resonance frequency of a CMUT is thus obtained by identifying the frequency at which the average membrane displacement versus frequency peaks, as illustrated in Figure 4b for an AC amplitude of 50 mV and various DC voltages. The distorted and asymmetric frequency response for a DC voltage of 17.5 V, compared to lower DC voltages, illustrates the effect of the non-linear electrostatic force [34]. The maximum DC voltage for which a symmetric frequency response can be obtained (17.5 V), while reducing the amplitude of the AC excitation signal (25 mV), occurs shortly before pull-in. When the CMUT transitions from conventional to collapse mode (25 V), the resonance frequency increases drastically, as the central portion of the membrane comes into contact with the substrate, effectively shortening the vibrating portion of the membrane [4].

**Figure 5:**
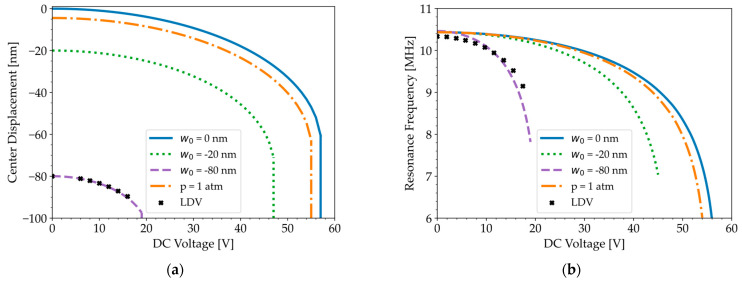
(**a**) Displacement–voltage curves, demonstrating close agreement between LDV measurements in air (symbols) and simulations for an initial center deflection of −80 nm (dashed line), while a pressure gradient of 1 atm acting on a flat membrane (dash–dotted line) causes a deflection of only −5 nm. (**b**) Spring softening effect simulated in COMSOL for various initial conditions (lines) compared to the resonance frequency measured in air with an LDV (symbols).

Figure 5b depicts the spring softening effect. The apparent spring constant or effective stiffness of the membrane is lowered by the increased non-linear electrostatic force [35], leading to a decrease in the resonance frequency from 10.33 MHz at DC = 0 V to 9.15 MHz at DC = 17.5 V. The displacement–voltage curve and spring softening effect in Figure 5a,b, respectively, demonstrate good agreement between the experimental data (symbols) and the simulations (dashed line) for an initial deflection of −80 nm. A maximum relative error of 5% near the pull-in voltage validates the COMSOL model for a CMUT with significant initial deflection. While the initial deflection has minimal effect on the resonance frequency (1%), it significantly reduces the pull-in voltage by 185%, compared to a pressure gradient of 1 atm, which only reduces the pull-in voltage by 3%.

### 3.2. C-V Curve and Mode Shapes: Experimental Results from LCR Meter and LDV

**Figure 6:**
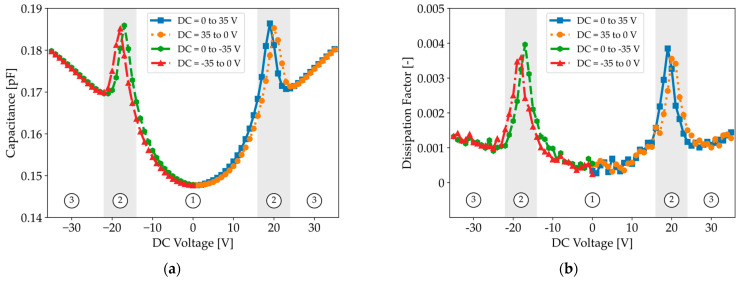
(**a**) Capacitance and (**b**) dissipation factor measured with an LCR meter (f = 100 kHz, AC = 0.5 V) when sweeping the DC voltage upward and downward for both polarities.

Experimental C-V measurements are performed using an LCR meter with a test frequency between 50 kHz and 1 MHz, which is well below the natural resonance frequency of the CMUT (10 MHz) to minimize dynamic effects that could distort the C-V curve. As shown in Figure 6a, the C-V curve exhibits minimal hysteresis when the DC voltage is ramped between 0 and ±35 V. Furthermore, the C-V curve reveals a transition region (zone 2) where the capacitance decreases as the voltage increases, while a sharp increase in capacitance is generally expected during the transition from the parabolic conventional region (zone 1) to the linear collapse region (zone 3).

In Section 2.2, we mentioned that the CMUT can be approximated as an ideal capacitor because the dissipation factor is very small. However, real-world capacitors exhibit a non-zero dissipation factor, representing the ratio of energy dissipated as heat to the energy stored in the capacitor. Figure 6b shows that the dissipation factor in the transition region (zone 2) is significantly higher than in the other two regions.

**Figure 7:**
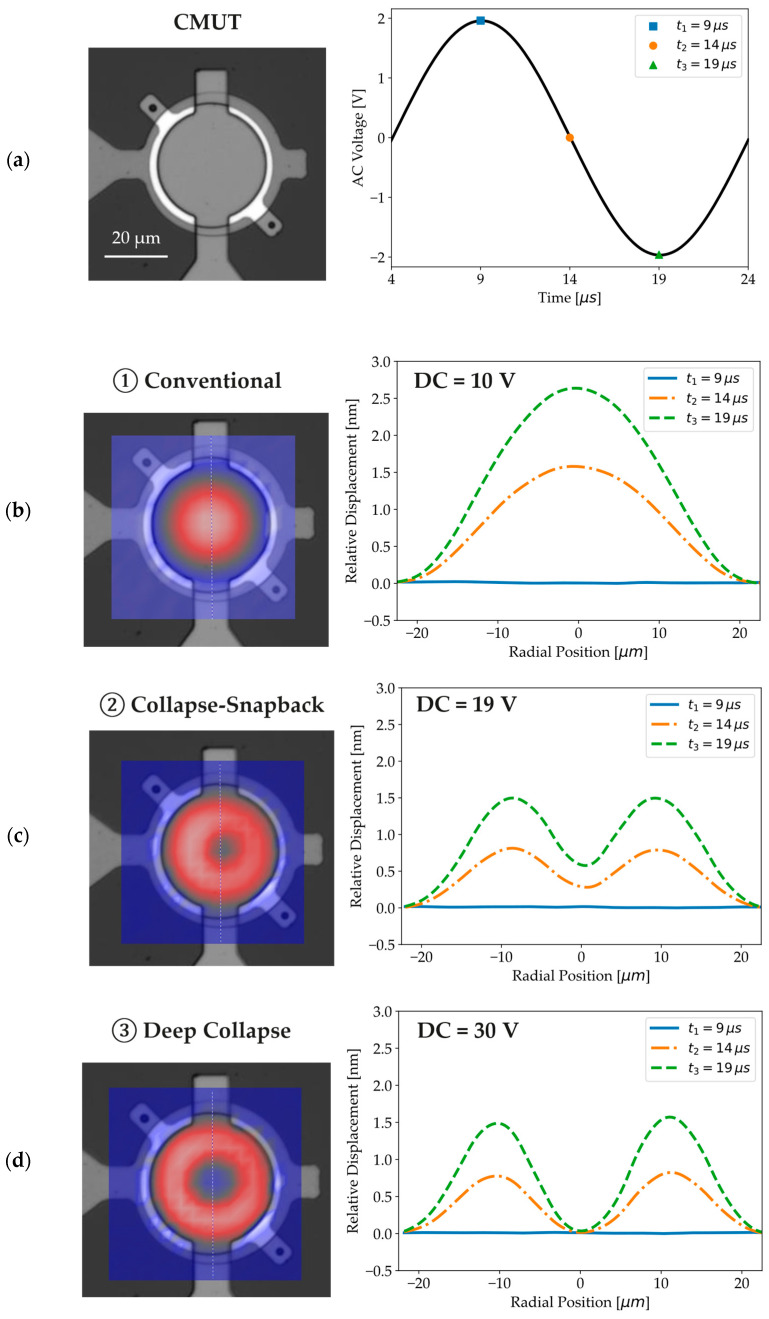
(**a**) AC voltage applied to a 40-um diameter CMUT to replicate the operating conditions of an LCR meter with a test frequency of 50 kHz. (**b**,**c**) Vibration mode and relative membrane displacement acquired with an LDV in the (**b**) conventional (DC = 10 V), (**c**) collapse-snapback (DC = 19 V), and (**d**) deep collapse (DC = 30 V) mode.

An LDV is used to record the CMUT vibration modes at a test frequency of 50 kHz, with an AC amplitude of 2 V and DC voltages of 10 V, 19 V, and 30 V, each corresponding to one of the three distinct regions of the C-V curve. Relative membrane displacements, extracted from vibration modes at key time steps highlighted in Figure 7a, reveal that the membrane center can move freely for DC voltages in zone 1 (Figure 7b), while its movement becomes increasingly restricted for higher voltages. In zone 2 (Figure 7c), the membrane center can still collapse and snap back, whereas it clearly remains in stiction for voltages in zone 3 (Figure 7d). Animations of the conventional, collapse–snapback, and deep-collapse modes, from which the relative displacements in Figure 7 have been extracted, can be visualized in the Supplementary Animations S2, S3, and S4.

The increased dissipation factor combined with the observed vibration modes supports the conclusion that the collapse–snapback phenomena in zone 2 are associated with significant energy loss.

**Figure 8:**
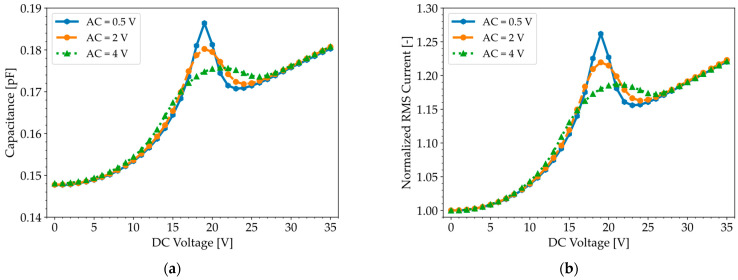
(**a**) Capacitance–voltage and (**b**) current–voltage curves measured with an LCR meter for a test frequency of 100 kHz and various AC amplitudes. Both plots exhibit a peak around the pull-in voltage (19 V).

The C-V and I-V curves displayed in Figure 8 reveal a linear relationship between the measured capacitance and RMS current, while also showing that the width and height of the peak are influenced by the amplitude of the AC test signal. Supplementary Figure S1 further demonstrates their independence from the test frequency (50 kHz–1 MHz), which is far below the CMUT’s natural resonance frequency. It is worth noting that we obtained similar C-V curves with the QSCV method. The similarity in the C-V curves for both characterization methods can be attributed to the fact that both the LCR meter and the parameter analyzer calculate capacitance based on current measurements.

### 3.3. C-V Curve: Simulation Results

**Figure 9:**
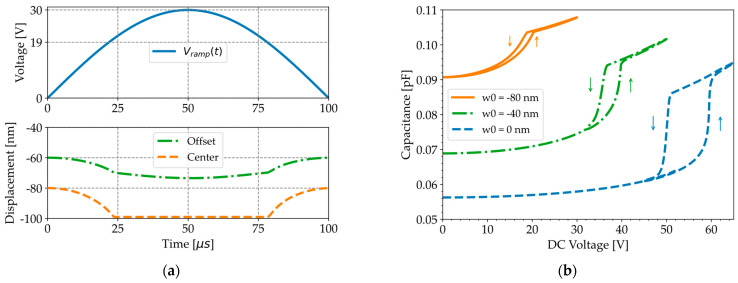
COMSOL simulations when the voltage is gradually ramped around pull-in and pull-out. (**a**) Displacement of the membrane center point (dashed line) and an offset point at half the membrane radius (dash–dotted line) for an initial center deflection of −80 nm and a ramp voltage varying between 0 and 30 V (solid line). (**b**) Capacitance–voltage curves for various initial deflections.

Pre-stressed time-dependent studies in COMSOL show that the peak in capacitance observed with the LCR meter near the pull-in voltage (Figure 8a) does not appear in the simulations when the DC voltage is ramped up with quasi-static loading (Figure 9b). The membrane center displacement (dashed line in Figure 9a) saturates when the ramp voltage (solid line in Figure 9a) surpasses the pull-in and pull-out voltages (19 V), as previously observed with the LDV (Figure 4b). However, the contact time for the LDV measurements is much shorter than for the simulations because the frequency of the applied sine wave is 50 kHz for the experiments and only 5 kHz for the simulations. The displacement of an offset point at half the membrane radius (dash–dotted line in Figure 9a) continues to increase beyond pull-in because the membrane edges are further pushed down by the increased electrostatic force. This effect is at the origin of the linear capacitance increase observed at higher voltages in Figure 9b.

The simulated C-V curves in Figure 9b show negligible hysteresis between pull-in and pull-out for an initial deflection of −80 nm, which is consistent with the experimentally measured C-V curves in Figure 6a. The contact radius after pull-in is larger than before pull-out because the contacting edges of the membrane are gradually released as the voltage is reduced, until the membrane snaps back completely. However, Table 5 shows that the difference in contact radius after pull-in and before pull-out decreases with larger initial deflection. As a result, the hysteresis between pull-in and pull-out becomes minimal.

**Figure 10:**
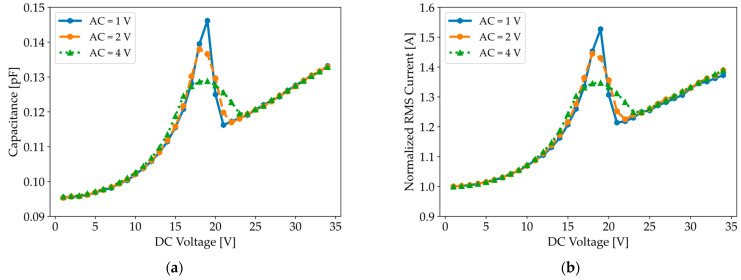
(**a**) Simulated capacitance–voltage curves and (**b**) current–voltage curves for an initial deflection of −80 nm, a test frequency of 5 kHz, and various AC amplitudes.

The next study consists of mimicking the operating conditions of the LCR meter by superimposing an AC voltage on a DC pre-stress voltage. The initial membrane position is obtained by ramping up the DC bias, as shown in Figure 9a, and evaluating the membrane deflection at the maximum voltage, which occurs at 50 µs. Subsequently, the membrane is excited with a 5 kHz AC test signal around its initial position. The capacitance is then calculated for a specific combination of AC and DC voltages based on impedance measurements, as presented in Equation (17). The simulated C-V and I-V curves, shown in Figure 10, reveal a peak in capacitance when the AC voltage is combined with a DC pre-stress voltage near the pull-in voltage, suggesting that the transient membrane displacement and additional current flow generated by the AC load cannot be ignored. The width and height of the peak depend on the AC amplitudes, as observed experimentally in Figure 8a.

**Figure 11:**
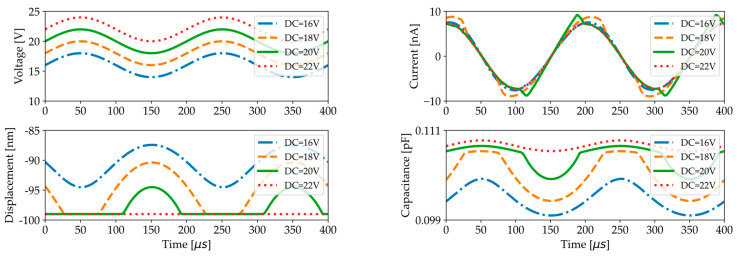
Simulated membrane center displacement, current, and capacitance as a function of time for a 5 kHz AC test signal with an amplitude of 2 V superimposed on various DC voltages.

The peak in RMS current and capacitance observed in Figure 10 can be explained by examining the time-dependent behavior of the membrane center displacement, current, and capacitance, as shown in Figure 11. When a 5 kHz AC voltage is superimposed on a DC voltage such that the total DC + AC voltage becomes closer to the pull-in voltage (19 V), the displacement becomes increasingly asymmetric due to the highly non-linear electrostatic force. For voltages oscillating around the pull-in voltage, we can see that collapse–snapback phenomena distort the current, while sinusoidal behavior is recovered when the membrane center remains in stiction for higher DC + AC voltages. Although the relationship between instantaneous capacitance and current is non-linear, a linear relationship exists between capacitance and the RMS current when measured under conditions of constant frequency, constant RMS voltage, and a relatively stable phase difference, as predicted by Equation (17). The higher the AC signal, the larger the DC range over which the membrane collapses and snaps back, hence the larger the DC range over which the current becomes distorted and a peak in capacitance is observed. However, the higher the AC signal, the smaller the peak in current or capacitance because the membrane center point remains in stiction for a longer time.

**Figure 12:**
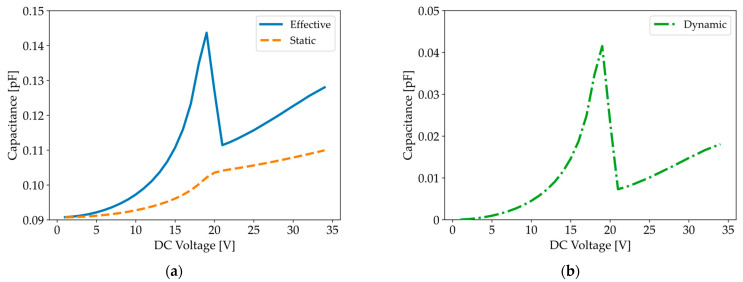
(**a**) Simulated C-V curve for an initial deflection of −80 nm, a test frequency of 5 kHz, and an AC amplitude of 1 V with the decomposition of the effective C-V curve (solid line) into static (dashed line) and (**b**) dynamic (dash–dotted line) capacitances.

When comparing quasi-static C-V simulations for an initial deflection of −80 nm in Figure 9b (solid line) with Figure 10a, we notice a greater increase in capacitance for devices subjected to an AC signal. The origin of this sharper increase can be demonstrated by decomposing the measured capacitance into static and dynamic parts, as introduced in Equations (19)–(21). Figure 12 clearly shows that the dynamic capacitance (dash–dotted line), which accounts for the transient membrane displacement, is responsible for the peak in the measured capacitance (solid line).

## 4. Conclusions

Through the development of advanced simulation and experimental methods, we proved that dynamic effects, such as collapse–snapback phenomena in CMUT, can significantly affect the effective capacitance–voltage curves of electrostatically actuated MEMS. First, using Laser Doppler Vibrometer measurements, we demonstrated that the C-V curve of a CMUT can be divided into three distinct operation regimes: conventional mode, collapse–snapback, and deep collapse. Next, we noticed that the peak in capacitance observed with the LCR meter near the pull-in voltage only appears in the COMSOL Multiphysics^®^ simulations when an AC signal is superimposed on a DC pre-stress, indicating that the transient membrane displacement and additional current flow induced by the AC load cannot be ignored. Additionally, we showed that collapse–snapback phenomena near the pull-in voltage alter the current waveform, leading to an apparent drop in the effective capacitance. Finally, we decomposed the measured capacitance into static and dynamic components, attributing the peak observed in the C-V curve to the dynamic component.

The COMSOL Multiphysics^®^ model provides a solid foundation for future studies on capacitive devices. In the realm of CMUT, we suggest extending the model with an acoustic domain to evaluate the transmit and receive sensitivity under various biasing conditions and environmental settings, such as in-water operation. Moreover, we propose investigating the impact of dielectric charging because this inherent reliability problem in capacitive devices can significantly affect capacitance–voltage measurements, hysteresis, and device performance. Finally, a comparison between the conventional, collapse–snapback, and deep-collapse modes in terms of electromechanical coupling efficiency, acoustic transmit–receive sensitivity, and reliability would provide valuable insights for enhancing the long-term performance of CMUTs.

## Figures and Tables

**Table 1:** Quasi-static capacitance–voltage (QSCV) measurement settings.

	DC Bias	Current Range	Voltage Step	Integration Time
**QSCV**	0 V–35 V	10 pA	ΔV = 0.5 V	ΔT = 1 s

**Table 2:** Design parameters for COMSOL simulations.

Geometrical Parameter	Variable	Unit	Value
Membrane radius	R	µm	20
Membrane thickness	$t_{mem}$	nm	1050
Top electrode thickness	$t_{top,elec}$	nm	250
Top insulator thickness	$t_{top,ins}$	nm	200
Sacrificial layer thickness	$t_{gap}$	nm	100
Bottom insulator thickness	$t_{bot,ins}$	nm	100

**Table 3:** Material parameters for COMSOL simulations.

Material Parameter	Variable	Unit	Si_3_N_4_	Au
Young’s modulus	E	GPa	210	70
Poisson’s ratio	ν	-	0.23	0.44
Density	ρ	kg/m^3^	3000	19,300
Dielectric constant	$\varepsilon_{i}$	-	7	-

**Table 4:** Resonance frequency and pull-in voltage for the analytical model, various COMSOL simulations, and experimental characterizations with an LDV and LCR meter.

	Resonance Frequency at DC = 0 V [MHz]	Resonance Frequency at DC = 17.5 V [MHz]	Pull-In Voltage [V]
Analytical model	10.33	10.18	56.38
COMSOL without initial deflection with full top-electrode coverage	10.19	10.04	55.68
**Relative error**	**1%**	**1%**	**1%**
COMSOL without initial deflection with 85% top-electrode coverage	10.43	10.30	57.12
COMSOL with initial deflection with 85% top-electrode coverage	10.46	8.70	19.60
Experimental characterization	10.33	9.15	19
**Relative error**	**1%**	**5%**	**3%**

**Table 5:** Simulation of the contact radius after pull-in and before pull-out for various initial deflections.

Initial Center Deflection	0 nm	−40 nm	−80 nm
Contact radius after pull-in	0.86 µm	0.56 µm	0.30 µm
Contact radius before pull-out	0.31 µm	0.20 µm	0.09 µm
Difference in contact radius	0.55 µm	0.36 µm	0.21 µm

## Data Availability

The original contributions presented in this study are included in the article/Supplementary Materials. Further inquiries can be directed to the corresponding author.

## Supplementary Materials

The following supporting information can be downloaded at: https://www.mdpi.com/article/10.3390/mi16020160/s1, Figure S1: C-V curves measured with an LCR meter for an AC amplitude of 1 V and test frequencies of 50 kHz, 100 kHz, and 1 MHz; Animation S2: LDV measurement in conventional mode (DC = 10 V); Animation S3: LDV measurement in collapse-snapback mode (DC = 19 V); Animation S4: LDV measurement in deep collapse mode (DC = 30 V).

## References

[1] Wang L.-F., Huang Q.-A., Han L., Huang Q.-A. (2018). RF MEMS Switch. Micro Electro Mechanical Systems.

[2] Park K.K., Oralkan O., Khuri-Yakub B.T. Comparison of conventional and collapse-mode CMUT in 1-D array configuration. Proceedings of the 2011 IEEE International Ultrasonics Symposium.

[3] Olcum S., Yamaner F.Y., Bozkurt A., Atalar A. (2011). Deep-collapse operation of capacitive micromachined ultrasonic transducers. IEEE Trans. Ultrason. Ferroelect. Freq. Contr..

[4] Pekař M., Dittmer W.U., Mihajlović N., van Soest G., de Jong N. (2017). Frequency Tuning of Collapse-Mode Capacitive Micromachined Ultrasonic Transducer. Ultrasonics.

[5] Merbeler F., Wismath S., Haubold M., Bretthauer C., Kupnik M. (2022). Ultra-Low-Voltage Capacitive Micromachined Ultrasonic Transducers with Increased Output Pressure Due to Piston-Structured Plates. Micromachines.

[6] Yamaner F.Y., Zhang X., Oralkan Ö. Fabrication of anodically bonded capacitive micromachined ultrasonic transducers with vacuum-sealed cavities. Proceedings of the 2014 IEEE International Ultrasonics Symposium.

[7] Park K.K., Oralkan O., Khuri-Yakub B.T. (2013). A comparison between conventional and collapse-mode capacitive micromachined ultrasonic transducers in 10-MHz 1-D arrays. IEEE Trans. Ultrason. Ferroelectr. Freq. Control..

[8] Munir J., Ain Q., Lee H.J. (2019). Reliability issue related to dielectric charging in capacitive micromachined ultrasonic transducers: A review. Microelectron. Reliab..

[9] Elata D., Bamberger H. (2006). On the Dynamic Pull-In of Electrostatic Actuators With Multiple Degrees of Freedom and Multiple Voltage Sources. J. Microelectromech. Syst..

[10] Seeger J., Boser B. Parallel-plate driven oscillations and resonant pull-in. Proceedings of the Solid-State Sensor, Actuator and Microsystems Workshop.

[11] Lin D.-S., Zhuang X., Wong S.H., Kupnik M., Khuri-Yakub B.T. (2010). Encapsulation of Capacitive Micromachined Ultrasonic Transducers Using Viscoelastic Polymer. J. Microelectromech. Syst..

[12] Park K.K., Kupnik M., Lee H.J., Oralkan O., Khuri-Yakub B.T. Zero-bias resonant sensor with an oxide-nitride layer as charge trap. Proceedings of the SENSORS, 2010 IEEE.

[13] Dew E.B., Kashani Ilkhechi A., Maadi M., Haven N.J.M., Zemp R.J. (2022). Outperforming piezoelectric ultrasonics with high-reliability single-membrane CMUT array elements. Microsyst. Nanoeng..

[14] Senturia S.D. (2001). Microsystem Design.

[15] Bayram B., Haeggstrom E., Yaralioglu G.G., Khuri-Yakub P. (2003). A new regime for operating capacitive micromachined ultrasonic transducers. IEEE Trans. Ultrason. Ferroelectr. Freq. Control..

[16] Huang Y., Haeggstrom E., Bayram B., Zhuang Z., Ergun A.S., Cheng C.H., Khuri-Yakub B.T. Collapsed regime operation of capacitive micromachined ultrasonic transducers based on wafer-bonding technique. Proceedings of the IEEE Symposium on Ultrasonics.

[17] Pekař M., van Nispen S.H.M., Fey R.H.B., Shulepov S., Mihajlović N., Nijmeijer H. (2017). A fluid-coupled transmitting CMUT operated in collapse mode: Semi-analytic modeling and experiments. Sens. Actuators A Phys..

[18] Olçum S. (2010). Deep collapse mode capacitive micromachined ultrasonic transducers. Dictoral Dissertation.

[19] Roy R., Farhanieh O., Ergun S., Bozkurt A. (2017). Fabrication of High-Efficiency CMUTs With Reduced Parasitics Using Embedded Metallic Layers. IEEE Sens. J..

[20] Xu T., Tekes C., Degertekin F. (2014). CMUTs with high-K atomic layer deposition dielectric material insulation layer. IEEE Trans. Ultrason. Ferroelectr. Freq. Control.

[21] Robertson J., Wallace R.M. (2015). High-K materials and metal gates for CMOS applications. Mater. Sci. Eng. R Rep..

[22] Keysight E4980A/AL Precision LCR Meter User’s Guide. https://www.keysight.com/us/en/assets/9018-05655/user-manuals/9018-05655.

[23] Keysight Impedance Measurement Handbook. https://www.keysight.com/us/en/assets/7018-06840/application-notes/5950-3000.pdf.

[24] Keysight Quasi-Static Capacitance Voltage Measurement Techniques. https://www.keysight.com/us/en/assets/7018-04703/application-notes/5992-0436.pdf.

[25] Engholm M., Pedersen T., Thomsen E.V. (2016). Modeling of plates with multiple anisotropic layers and residual stress. Sens. Actuators A Phys..

[26] El-Damak D.R., Hegazi E., Ragai H.F. Analytical design of circular CMUT cells in immersion. Proceedings of the 2010 IEEE International Ultrasonics Symposium.

[27] Leissa A.W. (1969). Vibration of Plates.

[28] Pursula P., Saarilahti J., Paul O., Viikari V. (2012). Analytical electromechanical model for CMUTs with multi-layered, non-uniform diaphragm. Proceedings MME 2012.

[29] Zhang W., Zhang H., Du F., Shi J., Jin S., Zeng Z. (2015). Pull-In Analysis of the Flat Circular CMUT Cell Featuring Sealed Cavity. Math. Probl. Eng..

[30] Olcum S., Senlik M.N., Atalar A. (2005). Optimization of the gain-bandwidth product of capacitive micromachined ultrasonic transducers. IEEE Trans. Ultrason. Ferroelectr. Freq. Control..

[31] Pull-In and Pull-Out Analysis of a Biased Resonator—2D. https://www.comsol.com/model/pull-in-and-pull-out-analysis-of-a-biased-resonator-2d-22141.

[32] Dutta S., Pandey A. (2021). Overview of residual stress in MEMS structures: Its origin, measurement, and control. J. Mater. Sci. Mater. Electron..

[33] Sheploak M., Dugundji J. (1998). Large Deflections of Clamped Circular Plates Under Initial Tension and Transitions to Membrane Behavior. J. Appl. Mech..

[34] Malatkar P., Wong S.F., Pringle T., Loh W.K. Pitfalls an engineer needs to be aware of during vibration testing. Proceedings of the 56th Electronic Components and Technology Conference.

[35] Yaralioglu G.G., Ergun S., Bayram B., Haeggstrom E., Khuri-Yakub P. (2003). Calculation and Measurement of Electromechanical Coupling Coefficient of Capacitive Micromachined Ultrasonic Transducers. IEEE Trans. Ultrason. Ferroelectr. Freq. Control..

